# Tracking the Evolution of Infrastructure Systems and Mass Responses Using Publically Available Data

**DOI:** 10.1371/journal.pone.0167267

**Published:** 2016-12-01

**Authors:** Xiangyang Guan, Cynthia Chen, Dan Work

**Affiliations:** 1 Department of Civil and Environmental Engineering, University of Washington, Seattle, WA, United States of America; 2 Department of Civil and Environmental Engineering, University of Illinois at Urbana-Champaign, Urbana, IL, United States of America; University of Florida, UNITED STATES

## Abstract

Networks can evolve even on a short-term basis. This phenomenon is well understood by network scientists, but receive little attention in empirical literature involving real-world networks. On one hand, this is due to the deceitfully fixed topology of some networks such as many physical infrastructures, whose evolution is often deemed unlikely to occur in short term; on the other hand, the lack of data prohibits scientists from studying subjects such as social networks that seem likely to evolve on a short-term basis. We show that both networks—the infrastructure network and social network—are able to demonstrate evolutionary dynamics at the system level even in the short-term, characterized by shifting between different phases as predicted in network science. We develop a methodology of tracking the evolutionary dynamics of the two networks by incorporating flows and the microstructure of networks such as motifs. This approach is applied to the human interaction network and two transportation networks (subway and taxi) in the context of Hurricane Sandy, using publically available Twitter data and transportation data. Our result shows that significant changes in the system-level structure of networks can be detected on a continuous basis. This result provides a promising channel for real-time tracking in the future.

## Introduction

Modern societies can be imagined as a set of intertwined networks relying on each other [1–3]. Critical infrastructures (e.g. transportation) provide essential services to human beings and on the other hand, aggregation of human responses, or mass responses, directly affects performance of the infrastructure system [4]. On a daily basis, these infrastructures along with the humans they serve and are affected by experience an ongoing evolutionary process during which failure and recovery of nodes and links lead to changes in the states of entire infrastructure and human systems [5–7]. This evolutionary perspective is particularly important, in the current and future times when external shocks such as climate change will bring to the world major disruptions of increasing frequencies and intensifying magnitudes [8, 9].

This evolutionary perspective has not been captured in the current literature on infrastructures. Nearly all adopt a static view on the infrastructure network, on top of which either steady or semi-steady flows are assumed (e.g. [10, 11]). Studies specifically focusing on major disruptions have only captured a limited number of stages among the entire lifecycle of a disruption (i.e. the pre-impact, impact, and post-impact stages) [12–14]. Similarly, studies on human responses are also limited to only a few stages and tend to focus on specific entities (e.g., emergency responders) [15–17]. Studies tracking human responses at a mass level on a continuous basis from pre-impact to during- and post-impact stages are rare.

In this paper, we identify a unique viewpoint that the evolutionary changes on the infrastructures and mass responses on a daily basis can be captured using publically available data, with a methodology that combines simple concepts and measurements developed from network science, statistics, and infrastructure studies. Using Hurricane Sandy as a case study, we capture topological changes in three networks on a daily basis by quantifying the temporal evolution of social-interaction patterns on the human network and changes in the flow patterns on two transportation networks, with a set of publically available data. Our result shows that significant changes on all three networks can be detected, providing a promising channel for real-time tracking in the future.

### Literature review

The perspective that infrastructure systems undergo constant changes at the network level is a new one for the majority of the infrastructure studies. These topological changes may be caused by the collapse of physical nodes and links or flows that are being re-distributed that ultimately affect the functions of a network. Many infrastructure studies (e.g. transportation) have long been studying flows, but most have not regarded flows as relating to network topologies; instead most have assumed a static network topology on which flows are steady or semi-steady [10, 11]. These studies are mostly interested in flows between sets of origins and destinations [14, 18, 19], as opposed to how flows at the network level may re-organize, which would provide crucial information on the evolution at the system level.

That networks can constantly go through an evolutionary process is not new in network science. However, most of the network science studies have focused on rather abstract, binary networks, as opposed to those that carry flows, like the real-world systems do [20]. While flow does not necessarily have a physical meaning on certain networks [21], it is conceivable that both the physical topology and flow shall play a role in influencing the function of infrastructure networks [22–25]. However, few existing performance metrics of infrastructure networks account for the topology and flow at the same time. One important goal of this study is to develop a metric that captures this evolutionary perspective and at the same time accounts for flow changes at the network level. We apply this metric to Hurricane Sandy to illustrate how system-wide infrastructure changes can be captured at the network level from before to during and after a major disaster.

Studies on human responses during or after a major disaster tend to focus on individuals, as opposed to mass responses. Individual- or household-based surveys are typically administered during or after a major disruption to understand human responses including actions taken and the associated reasons [26–29]. These surveys are often limited by their small sample sizes and restrictions of being retrospective [30, 31]. In recent years, the prevalence of social media tools has prompted a surge of studies using such data to analyze human responses during/after a major disruption [32–43]. Nearly all such studies focused on behaviors at the individual level, or more precisely, behaviors between specific agents such as government officials, emergency staff and celebrities. Furthermore, most of these studies are limited to a single during-impact stage and overlook the pre- and post-impact stages.

## Materials

### Study area and study period

This study is set in the backdrop of Hurricane Sandy, the second costliest hurricane ever recorded in the U.S. history [44], and within the area of and surrounding New York City (NYC), one of the most impacted regions by Sandy, with more than $50 billion in damages [45].

A circular area centered at the geographic center of NYC is selected as the study area. The radius is determined through a sensitivity analysis of the disaster-related ratio (DRR), which is simply the ratio between the number of disaster-related tweets and the number of general tweets within a geographical space, with respect to the radius (see S1 Fig). The result of sensitivity analysis suggests a radius of 39 km.

We define our study period to be the entire October and November of 2012. This study period is longer than the majority of the existing Sandy-relevant studies, which usually falls within from mid-October to mid-November in 2012 [46, 47].

### Data preparation on human responses

Human responses at a mass level are captured by tracking the social interactions that occur within the population that is affected by Sandy, given that social interactions constitute a critical dimension of human behaviors, especially in case of disasters [48, 49]. In particular, data from the online social media Twitter is used. We recognize that online social interactions may not be a representative sample of the entire ensemble of social interactions, but nonetheless they do provide insights into the patterns of more general social interactions, as social media platforms have increasingly replaced other means of communications [31, 50, 51].

We obtain the full set of geo-located tweets through an agreement between University of Washington and Twitter, Inc. It contains all tweets posted with a pair of geographic coordinates within our study area during the study period. This results in a total of 3,659,268 tweets. The snowball sampling method, a well-established sampling approach for online social networks [52] is applied to the dataset to generate 61 samples, one for each day in the October and November of 2012. We randomly select 0.1% of all Twitter users who were within the study area for each day as the seed. Then the user set is expanded by tracking the replying tweets from and to the seed users. Any Twitter user who posted at least one geo-located tweets (within the study area) replying to the seed users or who were replied to by the seed users are added to the user set. This procedure iterates on the updated user set until it reaches a depth of 6 from the seed users, following the rule of “six degrees of separation” [53]. All geo-located tweets posted by Twitter users in the user set, within the study area, and on the given day are included in the sample.

The online behaviors of Twitter users can be subject to the influence of multiple events. For example, the most likely competing event that affects people’s online behaviors during our study period is the 2012 presidential election. In order to ensure that the detected changes in online behaviors are mostly related to the effects of Hurricane Sandy, we categorize tweets into either disaster-related or not, and filter out those that show little relevance to a disaster or a potential disaster. We use the term “disaster-related tweets” to represent the tweets relevant to a (potential) disaster, and the term “general tweets” to represent the ensemble of both disaster-related tweets and non-disaster-related tweets. The method of tweet categorization is briefly described in the remaining of this section.

Prior to any tweet being identified as disaster related, there is only a general-tweet dataset. We randomly select 1,000 tweets from this dataset as the training dataset, and manually check if each of them is disaster-related or not, based on the rules set by Fresno et al. [54]. For the disaster-related tweets, we identify the words informative of their content (e.g. non-stop words), and put these words in our keyword set. This results in 99 keywords in the set after manually examining the 1,000 randomly-selected tweets.

Ideally, using all the 99 keywords to search the general-tweet dataset can capture all the disaster-related tweets. However, it may inflate the number of disaster-related tweets because some keywords can bring in tweets that are not disaster-related (which we call false positive). At the same time, using only a subset of the 99 keywords could result in missed disaster-related tweets (which we call false negative). A trade-off point between the false positive and false negative needs to be identified for the optimal set of keywords to be used.

To identify the trade-off point, we first calculate the probability at which each of the 99 keywords is able to correctly identify disaster-related tweets, that is, what proportion of the “disaster-related tweets” it identifies is indeed disaster-related. If the probability of correct categorization is larger than 95%, the false positive caused by this keyword is thought to be insignificant. And thus this keyword is included in our final keyword set. However, if the probability of correct categorization is smaller than 95%, false positive caused by this keyword is considerable and we move this keyword into a candidate set. This process ends up with 4 of the 99 keywords in the candidate set, namely “close”, “line”, “cancel” and “work”, which present a high probability (larger than 5%) of false positive categorizations.

We then examine every possible combination of the 4 keywords in the candidate set and plot the false-positive percentage and false-negative percentage when using a combination as the keyword set to search our training dataset (S2 Fig). False-positive percentage is calculated as the percentage of tweets that are categorized as “disaster-related” but are actually not disaster-related, and false-negative percentage is the percentage of tweets that are categorized as “non-disaster-related tweets” but are actually disaster-related. We identify the point where the summation of false-positive percentage and false-negative percentage is the lowest, which is shown as the point at index 11 in S2 Fig. The keywords in this combination are therefore added into our final keyword set. A list of keywords in the final keyword set can be found in S1 Text. We apply the final keyword set to the 61 snowball samples to search for disaster-related tweets. A tweet that contains at least one of the keywords in the final keyword set is a disaster-related tweet. In addition, a replying tweet to a disaster-related tweet is also deemed disaster-related, even if this replying tweet itself contains no keyword in the final keyword set.

As the last step of Twitter data preprocessing, we remove all the tweets posted by or replying to emergency responders or traditional news media, in order to focus on the interaction patterns among the general public. A list of removed Twitter users is included in S1 Text.

### Data preparation on infrastructure performance

To track performance of the infrastructure systems (transportation in this study), we integrate flows, or aggregates of individual human mobility patterns with network topologies, which focus on the physical layouts of the infrastructure networks [55, 56]. We collect data that contains information on flow and topology of the transportation networks in order to develop a comprehensive measure of infrastructure performance.

We obtain the subway ridership data from the New York Metropolitan Transportation Authority (NYMTA). It consists of daily tap-in counts for every subway station in NYC from October 1 to November 30 in the year of 2011 and 2012. This results in a 417 (number of subway stations) × 122 (number of days) data matrix.

The taxi trip records are obtained through a Freedom of Information Law (FOIL) request [47, 57], which is publicly available. We utilize particularly the locations of trip origins and the time each taxi trip starts in the dataset. A total number of 52,704,872 taxi trips in the October and November of 2011 and 2012 are analyzed.

## Methods

This study is approved by the Institutional Review Board of University of Washington, Seattle. All Twitter records and taxi trip records are anonymized and de-identified.

### Tracking changes in social interactions

We first construct a human network with nodes and links. In our case, a node represents a Twitter user, assuming that each user has only one Twitter account active during our study period. Links are based on replying relationships between Twitter users. An undirected link exists between two nodes (Twitter users) in either of the following two cases (S3 Fig): a) user A (B) posted at least one public tweet on a certain day and user B (A) replied to user A (B) on the same day; or b) user A and user B replied to each other on a certain day. We base links on replying relationship [42] instead of retweeting relationship [3, 42] because the replying relationship is more likely to reflect the interactions between Twitter users. We call this online social network “observed network” in order to distinguish it from null models.

**Fig 1:**
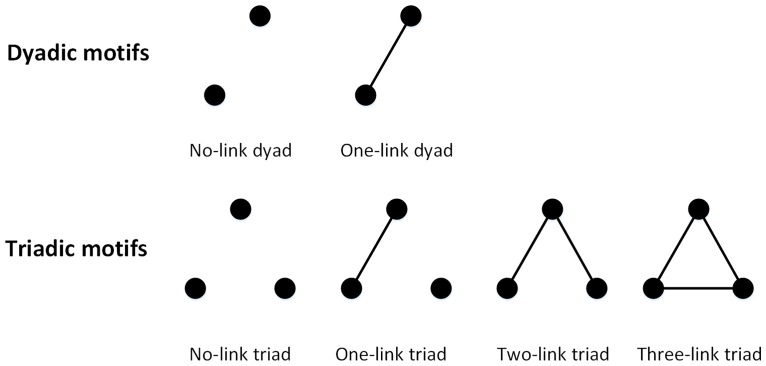
Possible dyadic and triadic motifs of an undirected network. The upper panel includes the two types of dyadic motifs: no-link dyads and one-link dyads; the lower panel lists the four types of triadic motifs: no-link triads, one-link triads, two-link triads and three-link triads. In this study, only no-link dyads, one-link dyads, two-link triads and three-link triads are considered.

Online-social-interaction patterns are characterized by two higher-order network structures [58]– dyadic motifs and triadic motifs, which are interconnection patterns (subgraphs) involving two nodes and three nodes, respectively [59]. Compared to global topological properties (e.g. average degree and clustering coefficient) employed by many studies (e.g. [31]), local topologies such as network motifs are more capable of capturing nuances in network structures [60] and are more associated to the functions of a network [61]. Fig 1 illustrates all possible patterns of dyadic and triadic motifs in an undirected network. For triadic motifs, we consider only patterns in which all three nodes are attached to edges [59], as a triadic motif with isolated nodes is equivalent to a dyadic motif. Therefore, two types of dyadic motifs and two types of triadic motifs, as we call no-link dyads, one-link dyads, two-link triads and three-link triads respectively, are of particular interest to us.

Conceptually, dyadic motifs and triadic motifs represent different dimensions of online social interactions. On one hand, dyadic motifs are related to the number of links in the network. For example, more one-link dyads suggest a higher number of links, or social interactions. On the other hand, triadic motifs are the natural generalizations of clustering coefficients [62], and therefore indicate whether a link tends to exist within a social circle or beyond. *In short, dyadic motifs capture the total amount of social interactions on Twitter, and triadic motifs reflect where those interactions are distributed*.

We employ a statistical approach [63] to evaluate the role of each of the four motifs in an observed network and develop measures of online-social-interaction patterns in the two above-discussed dimensions, as we call in this paper amount dimension and distribution dimension. The topology of a network is typically shaped by a number of correlated factors [59]. For example, increasing the number of dyads in a network is also likely to increase the number of triads in the network. Therefore, certain properties of the network must be controlled in order to examine the temporal changes of a network motif. This is implemented by introducing null models and z scores [60]. A null model is a network that resembles the observed network in a way that it shares certain controlled properties with the observed network while other properties are randomized. When looking at the presence of a network motif over time, null models act as benchmarks that normalize the number of this motif, so that the quantities measuring the presence of this motif in the network over time are at the same scale and comparable with each other. A commonly-used measurement of motif presence is z score. Statistically, a z score of a motif indicates how the presence of this particular motif deviates from what is predicted by the null models. It can be simply viewed as the normalized motif number with certain network properties controlled (see “Interpreting z scores” in S1 Text). In this paper, we use the value of ±3 as the threshold of z scores for testing whether the presence of a network motif is significant or not [59]. The threshold of ±3 (or equivalently 1% significance level) is chosen as it is most frequently used in existing network science studies [59, 63–65].

On each day of the study period, we generate 2000 null-model networks for the observed network on that day, and calculate the number of motifs in each null-model network. For a certain kind of motif *m*, the z score, which is a measure of the difference in the number of motif *m* between in the observed network and its expected value, is defined and calculated in Eq (1).

$$\begin{array}{c} z_{mi}=\frac{N_{mi}^{obs}-\left\langle N_{mi}^{null}\right\rangle }{\sigma (N_{mi}^{null})} \end{array}$$ (1)

where *z*_*mi*_ represents the *z* score of motif *m* on day *i*, $N_{mi}^{obs}$ is the number of motif *m* in the observed network on day *i*, and $\langle N_{mi}^{null}\rangle$ and $\sigma (N_{mi}^{null})$ are the average number of motif *m* and its standard deviation respectively in the null models corresponding to day *i*.

We apply two types of null modes as the benchmark model for investigating dyadic motifs and triadic motifs, respectively. First, the scale-free networks serve as null models for evaluating the dyadic motifs [66] based on the consideration that networks of social interactions present the scale-free property [67]. The controlled property in this case is the degree distribution. We fit a power-law degree distribution for each observed network and construct null-model networks that follow the same power-law degree distribution. We show in supporting information that the scale-free networks perform better as null models than the commonly employed exponential random graph (see “Evaluating dyadic motifs with exponential random graphs” in S1 Text). And next, we employ the configuration models as null models for evaluating the triadic motifs [68], and control the node degrees and dyadic structures in the null models to stay the same as in the observed network.

The calculated z scores are indicators of online-social-interaction patterns. To detect changes in social interactions, we compare the daily z scores against the average z score during the pre-impact phase (see results section) and calculate the z score of deviation using Eq (2).

$$\begin{array}{c} \Delta z_{mi}=\frac{z_{mi}-\left\langle z_{m,pre}\right\rangle }{\sigma (z_{m,pre})} \end{array}$$ (2)

where *z*_*mi*_ is the z score of motif *m* on day *i*, and 〈*z*_*m*,*pre*_〉 and *σ*(*z*_*m*,*pre*_) are the average z score of motif *m* during the pre-impact phase and its standard deviation respectively. Since z scores are normalized, Δ*z*_*mi*_ is also normalized, and its significance can be statistically tested.

### Tracking infrastructure network changes

We construct the subway network as an undirected network. Each subway station in NYC is viewed as a node, with the daily station-level ridership as a node attribute attached to it. A link exists between two nodes if the following two conditions are both satisfied: first, there is a subway line segment connecting these two nodes; and second, there are no other subway stations on this subway line segment.

To eliminate the effects of inter-station discrepancy and weekly patterns, we benchmark subway ridership in our study period on the corresponding 2011 ridership. For every station on each day of week during the study period, the benchmark is calculated as the average ridership of this station over all the same day of week in October and November of 2011. We correspond a 2012 ridership with a benchmark if they are of the same subway station, and on the same day of week. For example, the benchmark for the Times Square station on Monday will be the average ridership of Times Square station over all Mondays in the months of October and November of 2011. The ridership to use for analysis is the difference between a 2012 ridership and its corresponding benchmark.

To construct the taxi network, we divide NYC into small regions based on a Voronoi diagram of subway stations. The principle is that an arbitrary point in a region is the closest to the subway station in this region compared to other subway stations. Each of these small regions is viewed as a node in the taxi network, with the daily number of taxi pick-ups attached to it. An undirected link exists between two nodes if these two regions share borders. A similar process as for the subway ridership is applied to the number of taxi pick-ups to benchmark it upon the 2011 day-of-week average. The benchmarked number of taxi pick-ups is used for further analysis.

We then construct a flow-based assortativity measure [69] to track changes in distribution patterns of flows in the NYC subway network and taxi network respectively. This flow-based assortativity can account for flows both at the node level and at the network level, which together characterize overall performance of the infrastructure networks. A quantity, *e*_*xy*_, is defined to be the fraction of links in the network that connect two nodes with ridership/taxi pick-ups *x* and *y*, respectively. In our case, as duplicate links are not permitted, *e*_*xy*_ will always be 1/L where L is the total number of links in the subway/taxi network, if there exists a link between two nodes with ridership/taxi pick-ups *x* and *y*. The assortativity coefficient of a subway/taxi network is calculated using Eq (3).

$$\begin{array}{c} r=\frac{\sum_{xy}xy\left(e_{xy}-a_{x}b_{y}\right)}{\sigma_{a}\sigma_{b}} \end{array}$$ (3)

where *a*_*x*_ and *b*_*y*_ are, respectively, the fraction of links that start and end at nodes with ridership/taxi pick-ups *x* and *y*, calculated as *a*_*x*_ = ∑_*y*_ *e*_*xy*_ and *b*_*y*_ = ∑_*x*_ *e*_*xy*_, and *σ*_*a*_ and *σ*_*b*_ are the standard deviations of *a*_*x*_ and *b*_*y*_. Since the subway network and taxi network are both undirected, *a*_*i*_ = *b*_*i*_ where *i* represents a node with ridership/taxi pick-ups *i*. This flow-based assortativity measure can be interpreted as the correlation between the ridership/taxi pick-ups of any two connected nodes in the subway/taxi network, and the value of r ranges from -1 to 1. Increasing *r* of a subway/taxi network suggests that nodes in the network are increasingly likely to be connected with other nodes of similar ridership/taxi pick-ups; and decreasing *r* means nodes tend to be connected with others of different ridership/taxi pick-ups.

Similarly as with social-interaction patterns, we then compare the calculated, daily flow-based assortativity for the subway and taxi network against the average assortativity calculated for the pre-impact phase (from October 1 to October 27, 2012) using Eq (4).

$$\begin{array}{c} z\left(r\right)=\frac{r-\langle r_{pre}\rangle }{\sigma (r_{pre})} \end{array}$$ (4)

where *r* is the assortativity coefficient of the subway/taxi network on a certain day, and 〈*r*_*pre*_〉 and *σ*(*r*_*pre*_) are the average assortativity coefficients and its standard deviation respectively over the pre-impact phase. The normalized assortativity is a measure of the deviation of subway/taxi usage on any given day from the pre-impact norm, and thus reflects how the infrastructure performance has changed.

## Results

### Evolution of mass responses

**Fig 2:**
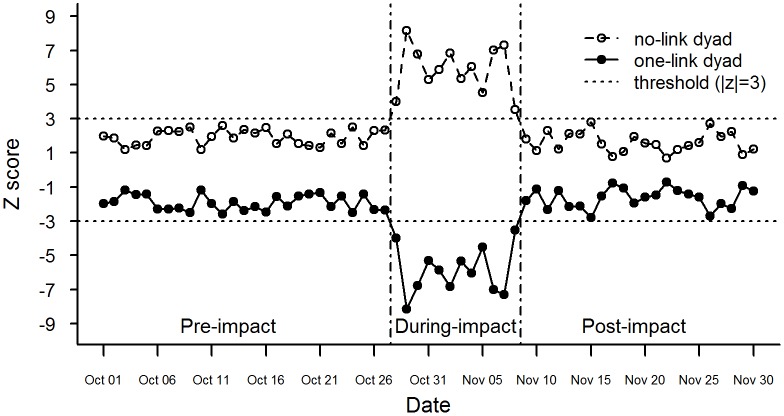
Temporal trajectory of dyadic motifs. The z score of a dyad measures the deviation of the number of this dyad from its expected number predicted by the null models. Its magnitude indicates the importance of a dyad in determining the structure of the online-social-interaction network.

**Fig 3:**
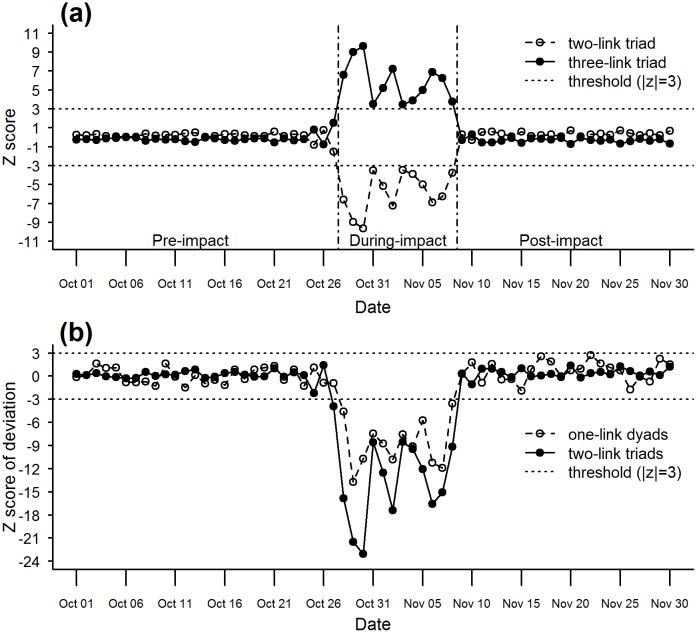
Temporal trajectories of triadic motifs. (a) shows the change of the z scores over time for the number of two-link triads and three-link triads respectively. (b) shows the everyday deviation of the z score from the average pre-impact z score. The deviation reflects the difference in online-social-interaction patterns on a particular day from pre-impact patterns.

The z scores of dyadic motifs and triadic motifs over time are presented in Figs 2 and 3a, respectively. The z scores of one-link dyads and no-link dyads are strictly symmetric around the line *z* = 0, as with the z scores of two-link triads and three-link triads. Transitions between the insignificant phase (when *z* ∈ (−3,3)) and the significant phase (when *z* ∈ (−∞,−3]∪[3,∞)) can be observed for both the dyadic motifs and triadic motifs. All four types of motifs have insignificant z scores from October 1 to October 27, abruptly shift to the significant phase on October 28 and remain while fluctuating in this phase until November 9 when they return to the insignificant phase simultaneously. Interpreting this result requires noting the signs of z scores (see “Interpreting z scores” in S1 Text). For the dyadic motifs, the z scores of no-link dyads are always positive while that of one-link dyads consistently negative. It signifies that no-link dyads tend to be overrepresented and one-link dyads underrepresented in the online-social-interaction network. The insignificant z scores before October 28 and after November 8 indicate that organization of the online-social-interaction network is similar to a scale-free network during this period. The network organization deviates from the scale-free model between October 28 and November 8, marked by the significant z scores. In particular, the amount of online social interactions (equivalently the number of links in the network) is significantly higher than the scale-free model can predict between October 28 and November 8, given also the (significant) positive z scores for the no-link dyads and (significant) negative z scores for the one-link dyads during this period. This result is validated by existing research on disaster-oriented Twitter usage: during a disaster, people tend to reduce usage of replying tweets [36], leading to lessened online social interactions as shown in our quantification of social interactions of replying behaviors on Twitter. Our result is also consistent with findings for mobile phone users, among whom decreased amount of social interactions prior to a large event is observed [33], given that there exists a warning or notice before the event. In the case of Hurricane Sandy, official disaster warning was issued on October 27, 2012 by National Hurricane Center (NHC) [70].

For triadic motifs, the temporal evolutions from the insignificant phase to the significant phase and back to the insignificant phase in the disaster lifecycle share commonalities with the dyadic motifs. Before October 28 and after November 8, the z scores are close to zero, showing that the triadic structure of the online-social-interaction network is almost identical to that of a configuration model with the same node degree sequence. The period between October 28 and November 8, characterized by significant negative z scores for two-link triads and significant positive z scores for three-link triads, suggests that from October 28 to November 8, there are significantly more three-link triads and less two-link triads with respect to those in the configuration models which serve as benchmarks. In other words, compared to the period before October 28 or after November 8 when the triadic structure is similar to the configuration models, the period between October 28 and November 8 witnesses an online-social-interaction network that is more likely to form three-link triads (and thus less likely to form two-link triads). This organizing principle could eventually lead to emergence of closely connected clusters where everyone interacts with each other (a.k.a. social circles or communities). Our finding is in tune with existing studies at individual level or for specific social groups and communication means, which suggest disaster-time social interactions becoming repeating, redundant, close-knit and localized [15, 33, 50, 71].

Based on whether the z scores are significant or not, the temporal evolutions of both the dyadic and triadic motifs divide the life cycle of Hurricane Sandy into three phases: the pre-impact phase (before and on October 27), during-impact phase (October 28 to November 8), and post-impact phase (on and after November 9). The during-impact phase is characterized by significant z scores for the four types of motifs, which implies that the amount of online social interactions decreases and densely connected communities emerge in the online-social-interaction network of the general public. These patterns are not obvious in the pre- and post-impact phase due to insignificant z scores.

To detect the temporal changes in online-social-interaction patterns, we apply the life-cycle perspective to the z scores of dyadic and triadic motifs. Because of the symmetry between no-link dyads and one-link dyads and between two-link triads and three-link triads, without loss of generality we select one-link dyads and two-link triads to represent the dyadic motifs and triadic motifs, respectively. Changes in online-social-interaction patterns can be evaluated as the deviations from pre-impact online-social-interaction patterns, or mathematically speaking, deviations from the expected z score of the corresponding motif in the pre-impact phase (see methods section). In Fig 3b, such deviations become significant during the disaster for both dyadic motifs and triadic motifs, but with different initial dates: the dyadic patterns enter the significant phase on October 28, while the triadic patterns are first significant one day earlier on October 27. In other words, evolutions of the structures of online-social-interaction networks occur in its triadic structures prior to in its dyadic structures. Given that triadic structures are of higher order than dyadic structures [59], we argue they could be more sensitive to disruptions in the network. As a result, triadic motifs are able to detect and predict such disruptions whereas lower-order network structures can not. In fact, it was found with other types of networks (e.g. financial networks) that triadic motifs could be a precursor for network collapse while dyadic motifs start to change only when the collapse takes place [63]. In terms of online-social-interaction patterns, this finding shows that under disaster situation formation of (new) online communities happens before the amount of online social interactions drops, suggesting possible causal relations between the two patterns.

### Evolution of infrastructure network changes

**Fig 4:**
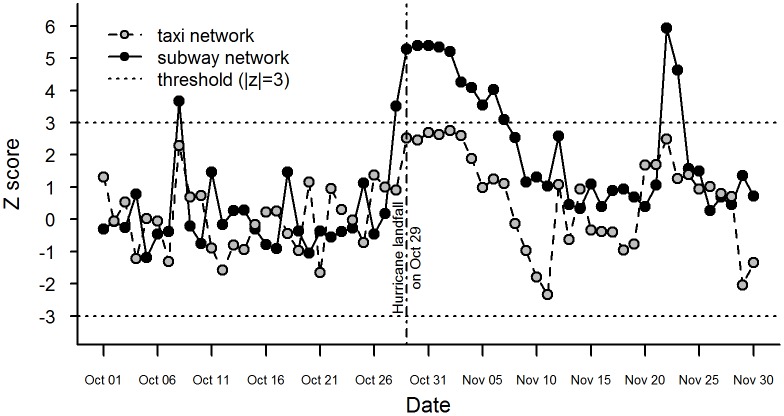
Temporal evolution of infrastructure networks. The z score indicates deviation from regular human mobility patterns (assumed to be the pre-impact patterns), a metric of infrastructure performance.

The normalized assortativity for the subway network and taxi network (Fig 4) show a similar overall trend: while fluctuating, they both go upwards before October 29, peak from October 29 to November 2, and decrease afterwards. Their differences lie in whether deviations from pre-impact norms are significant or not. For the subway network, z scores are significant from October 28 to November 7. This is an indicator that during this period, subway stations with similar ridership tend to be geographically clustered, that is, stations with high ridership tend to be close to other stations with high ridership, and the same for stations with low ridership. Given that subway ridership represents the number of people entering a station, this result implies that trip origins of subway riders tend to be geographically clustered, which is consistent with previous observations [72]. Taxi operation changes in a similar overall trend, but the significance of changes in the taxi network cannot be asserted with the use of the threshold of ±3. If the threshold of ±2 were used, it would be significant from October 29 to November 3. The insignificance is likely due to the fact that for the entire study period taxi trips are highly concentrated around a few origins (e.g. airports), leaving zero to few trips for most of the regions in our study area. The subway, on the other hand, is a major travel mode for nearly half of New Yorkers [73], and demonstrates comparatively more balanced usage in space than taxies. The unusual flow patterns observed from October 28 to November 7 in the subway network also indicate much severer disruptions to the subway system compared to the taxi operations which has only modest changes that are insignificant. Based on our flow-based measure, it is concluded that the subway infrastructures suffer an overall greater impact from Hurricane Sandy than the taxi infrastructures do.

We can piece together a story on how the general public as a whole responded to Sandy and how these responses lead to changes in the flows reflected on the infrastructure networks, by combining the findings on the infrastructure and human responses. The story begins with issuance of the official warning for Hurricane Sandy by National Hurricane Center (NHC) on October 27, 2012. Subtle changes in online-social-interaction patterns were immediately happening following the warning though the changes only became obvious about one day later. Nevertheless, the evolutions of online-social-interaction patterns on October 27 imply that the general public became sufficiently aware of the disaster and people’s behaviors start to differ from normal conditions after the warning issuance. Abnormal infrastructure performance emerges on the following day of October 28, marked by significantly different flow patterns from pre-impact norms. The mass response to the disaster by the general public and the emergent operational state of the subway infrastructures last for the next 13 and 11 days and die down on November 9 and November 8, respectively.

## Discussion

Using a set of publically available data, we have developed a set of novel methods to track mass responses and infrastructure network changes on a daily basis from before, to during and after a major disaster. The study was motivated by a number of factors relating to multiple disciplines, including the lack of acknowledgement that infrastructures are time-varying systems at a fine temporal scale in infrastructure studies, the absence of flows in most of the network science studies, and the mere focus on individual responses following a major disaster, as opposed to mass responses. To capture network-level changes in infrastructure systems, we construct a flow-based assortativity measure that captures flow-based topological changes on two networks: the subway network and the taxi network. We also developed measures involving dyadic and triadic motifs to track changes in human responses at a mass level. Our result shows that significant changes at the network level can be detected on a continuous basis for both the human network and infrastructure networks. This result provides a promising channel for real-time tracking in the future.

It is also hoped that this study will lead to the converging of two largely independent disciplines: infrastructure studies and network science. Infrastructure systems are the mainstay of the nation’s economy and well-being: they provide essential services that touch upon everyone’s life, and every sector that matters to the society. Though studies of infrastructure systems have long viewed them as networks, they have always viewed the underlying networks as static and fixed. The perspective that networks may be evolutionary over time is long amiss. Bringing in this new perspective will likely result in transformative changes in many infrastructure studies in terms of how networks are conceptualized, what are the important research questions and how the methodologies may be reformulated. On the other hand, studies in network science have long recognized this network-evolutionary perspective, though most of the networks studied are abstract and do not carry flows. Bringing flows into these studies and understanding how networks function in the real world would be a very important next step to further advance the state of the art.

The study also has important implications for disaster prediction, response, recovery, and mitigation. We discover that higher-order network structures (e.g. triadic motifs) are capable of detecting the impact of a disaster on the mass population at an early stage even before the impact is fully unfolded and clearly alters the population’s behaviors (e.g. reduced links, or dyadic motifs in the social interaction network). This finding has both theoretical and practical significance: on one hand it contributes greatly to reaching a more universal conclusion in network science with respect to identifying early-warning signals of system collapse [74]; and on the other hand higher-order network structures provide a promising channel for predicting the impact of a disaster or large event on the general public ahead of time in the pre-impact phase. Two other major findings of our study—that social interaction networks and infrastructure networks both experience abrupt evolutions in their topologies following a disaster warning and that such evolutions can be captured in real time using publicly available data—shall equip emergency responders with the knowledge, resources (e.g. data) and tools (e.g. methods) to monitor and respond to these changes in a real time fashion. These capabilities are critically important in the response and recovery stage. An additional insight shows the potential power of social media that may be leveraged to increase public awareness that likely precedes public actions. These efforts will likely enhance mitigation prior to a disaster and expedite recovery in the wake of it.

## Supporting Information

S1 Text Additional technical details. This file further explains the method used in this manuscript and provides justification for the choice of null models. It also lists the keywords used for tweet categorization and removed Twitter users that represent emergency responders and traditional news media.
(PDF)

S1 Fig Sensitivity analysis to determine study area. The plot shows how DRR changes with increasing distance from the geographic center of NYC. The fitted curve reaches its “elbow” point at the distance of 39 km.
(TIF)

S2 Fig Determining the optimal keyword combination. The two criteria, false negative and false positive both need to be minimized. While the two objectives can not be reached at the same time, we select the point where the summation of the two is minimized (at index 11). As a result, the keywords “close”, “line” and “cancel” are selected.
(TIF)

S3 Fig Constructing social media network from tweets. Node A and node B represent two Twitter users. Stubs without arrows represent public tweets, and lines with arrows represent replying tweets. An undirected link exists between A and B if a) A replied to B or vice versa; or b) A and B replied to each other. If c) no replying tweet exists between A and B, A and B are not considered connected in the social media network.
(TIF)

S4 Fig Z scores of dyadic motifs using Erdős–Rényi random graphs as null models. Similar with scale-free networks, the controlled property in this case is the degree distribution. The differences are that the degree distribution is assumed to be exponential in a Erdős–Rényi random graph and power-law in a scale-free network.
(TIF)

S1 Data NYC Subway ridership. This file contains the station-level ridership (tap-in counts) for the 417 stations in New York City, from October 1 to November 30 in 2011 and 2012 respectively.
(CSV)

S2 Data Twitter data. This file contains the Twitter data used in this study. Tweets’ IDs and users’ IDs are anonymized by randomly replacing the numbers in the IDs with letters.
(ZIP)
