# Supplementary material for: Tracking the Evolution of Infrastructure Systems and Mass Responses Using Publically Available Data
Source: PLoS One. 2016 Dec 1;11(12):e0167267. doi: 10.1371/journal.pone.0167267 (PMC5132226; doi:10.1371/journal.pone.0167267)
Supplement: S1 Text — This file further explains the method used in this manuscript and provides justification for the choice of null models. It also lists the keywords used for tweet categorization and removed Twitter users that represent emergency responders and traditional news media. (PDF) [file pone.0167267.s001.pdf]

Additional technical details

Interpreting z scores. The most straightforward interpretation of a z score, as is conveyed by how it is calculated, is the deviation of a quantity (e.g. the number of motifs) from the expected value of this quantity predicted by null models. The deviation is normalized by the standard deviation of this quantity in null models. Therefore, the threshold of ±3 can be used to test the significance (at 0.01 significance level) of the deviation, or in other words whether the observed network is significantly different from null-model networks in term of a certain structural property. By using the threshold of ±3, it is assumed that in null-model networks, the value of a certain structural property has a very small probability (<0.01) of being larger than 3 or smaller than -3. It is thus not necessary that the null model follows normal distribution.

Considering that null-model networks are randomized versions of the observed network and that a z score is the standardized deviation from null models, a z score also indicates whether the network is randomly organized in terms of a certain structure, if the appropriate null model is applied [1]. For example, a low z score of triadic motifs means the triadic property of the observed network is similar to null-model networks that have randomized triadic structures, and thus the triadic structures of the observed network tend to be randomly organized. Furthermore, since the triadic structures of the observed network is randomly organized and thus does not reflect any mechanism (other than being totally random) underlying the formation of the observed network, it is equivalent to state that the triadic structures convey little additional information about the structure of the observed network compared to what is controlled by null models.

Statistically, a z score also indicates to what extent the null models can explain the variations in the presence of a network motif. A low z score implies that the controlled properties in null models explain most of the variations in the presence of a network motif. Typically this means any network property of a certain order will also be explained by the controlled lower-order properties in the null models. For example, a low z score of triadic motifs indicates that the majority of the variations in the number of

triadic motifs are explained by the number of dyadic motifs (or even lower-order topological properties, depending on what is controlled by the null models). Furthermore, it implies the organization of triadic structures (which involve three nodes) is completely predicted by dyadic structures (which involve two nodes).

Evaluating dyadic motifs with exponential random graphs. In this paper, we have employed scale-free networks as null models in looking at the evolution of dyadic motifs. In existing research practice, a more commonly used null model for evaluating dyadic structures is the Erdős–Rényi random graph [2], whose degree distribution follows an exponential distribution (if the network size is sufficiently large) instead of a power-law distribution as in a scale-free network. Following this convention, we generate Erdős–Rényi random graphs as null models for dyadic motifs and calculate the corresponding z scores (S4 Fig).

While the overall trends of z scores are similar between using Erdős–Rényi random graphs as null models and using scale-free networks as null models, the magnitudes of their z scores are not at the same level. Using Erdős–Rényi random graphs always results in z scores larger in absolute values than using scale-free networks for the same observed network, and z scores under Erdős–Rényi random graphs can be significant even during non-disaster times (e.g. in the pre-disaster phase). As z scores indicate the deviations in dyadic structures from as predicted by null models, a smaller z score suggests that the null models resemble the observed network to a larger extent. Consequently, the degree distribution of the observed network is closer to a power-law distribution than to an exponential distribution, which is consistent with findings in network science. Considering that the objective of null models for dyadic motifs is to control the degree distribution to be the same as the observed network, the scale-free network is considered a better null model for dyadic motifs than the Erdős–Rényi random graph in the context of our study.

A list of keywords in the final keyword set: aftermath, alert, assist, batter, blackout, Bloomberg, cancel, catastrophe, charge, close, concern, ConEdison, coop, crane, cyclone, damage, danger, dark, destroy, destruct, disaster, donate, electric, emergency, evacuate, fearless, fema, fine, fire, floater, flood, food, forecast, gallon, gas, generator, heat, help, hurricane, impact, infrastructure, Katrina, landfall, light, line,

mess, mta, nor'easter, normal, outage, panic, plug, power, prep, prepare, pump, queue, rain, ready, recover, rescue, response, restore, restrict, safe, Sandy, scare, scary, service, shelter, shortage, shower, shut, snowfall, sound, status, store, storm, strand, stuck, submerge, suffer, supplies, supply, surge, tank, tide, transit, tree, truck, tsunami, victim, volunteer, warn, water, weather, wind, zone.

A list of removed Twitter users (in parenthesis are their Twitter account names): Governor of NY (@NYGovCuomo), Governor of NJ (@GovChristie), NYC Major (@MikeBloomberg), NYC Mayor's Office (@NYCMayorsOffice), National Weather Service (@NWS and @NWSNewYorkNY), National Oceanic and Atmospheric Administration (@NOAA), Federal Emergency Management Agency (@fema and @femaregion2), American Red Cross (@RedCross), U.S. Coast Guard (@USCG and @uscoastguard), National Guard (@USNationalGuard), U.S. Senators (@CoryBooker, @SenGillibrand and @MartyMarkowitz), NYC Council Members (@DanGarodnick and @JimmyVanBramer), utility operators (@ConEdison and @PSEGdelivers), public transit operators (@MTA, @ NJTRANSIT and @NYCTBus), ABC News (@ABC, @ABCWorldNews and @ABC7NY), CBS News (@CBSNews), CNN (@cnnbrk), NBC (@NBC and @NBCNewYork), the New York Times (@NYTMetro), Huffington Post (@HuffingtonPost), New York Daily News (@NYDailyNews), NY1 News (@NY1), the Weather Channel (@weatherchannel), WNYC (@WNYC).

Reference

1. Maslov S, Sneppen K, Zaliznyak A (2004) Detection of topological patterns in complex networks: correlation profile of the internet. Physica A: Statistical Mechanics and its Applications 333: 529-540.
2. Squartini T, van Lelyveld I, Garlaschelli D (2013) Early-warning signals of topological collapse in interbank networks. Scientific reports 3.
