# Supplementary figures and images for: Tracking the Evolution of Infrastructure Systems and Mass Responses Using Publically Available Data
Source: PLoS One. 2016 Dec 1;11(12):e0167267. doi: 10.1371/journal.pone.0167267 (PMC5132226; doi:10.1371/journal.pone.0167267)

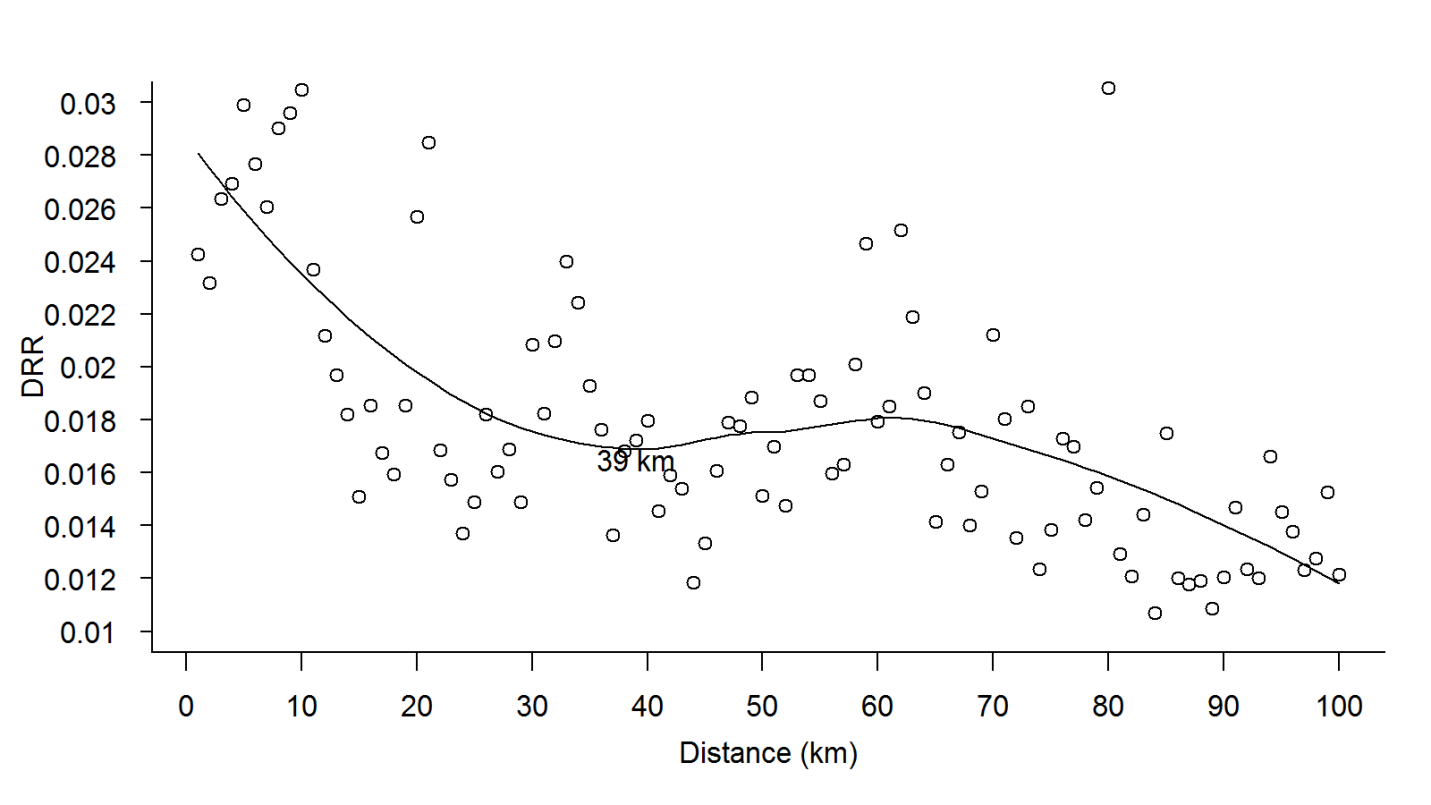

Supplement: S1 Fig — The plot shows how DRR changes with increasing distance from the geographic center of NYC. The fitted curve reaches its “elbow” point at the distance of 39 km. (TIF) [file pone.0167267.s002.tif]

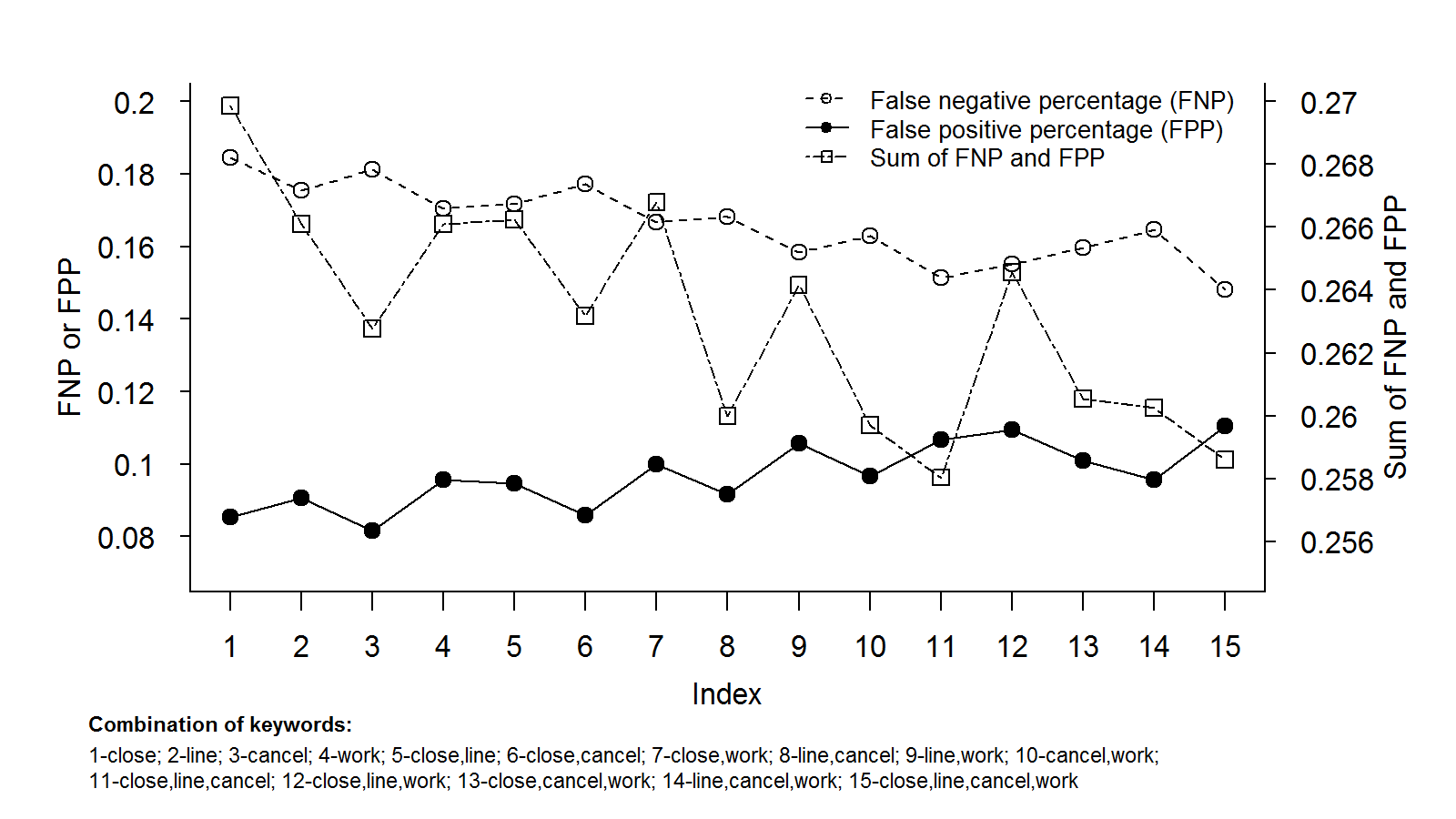

Supplement: S2 Fig — The two criteria, false negative and false positive both need to be minimized. While the two objectives can not be reached at the same time, we select the point where the summation of the two is minimized (at index 11). As a result, the keywords “close”, “line” and “cancel” are selected. (TIF) [file pone.0167267.s003.tif]

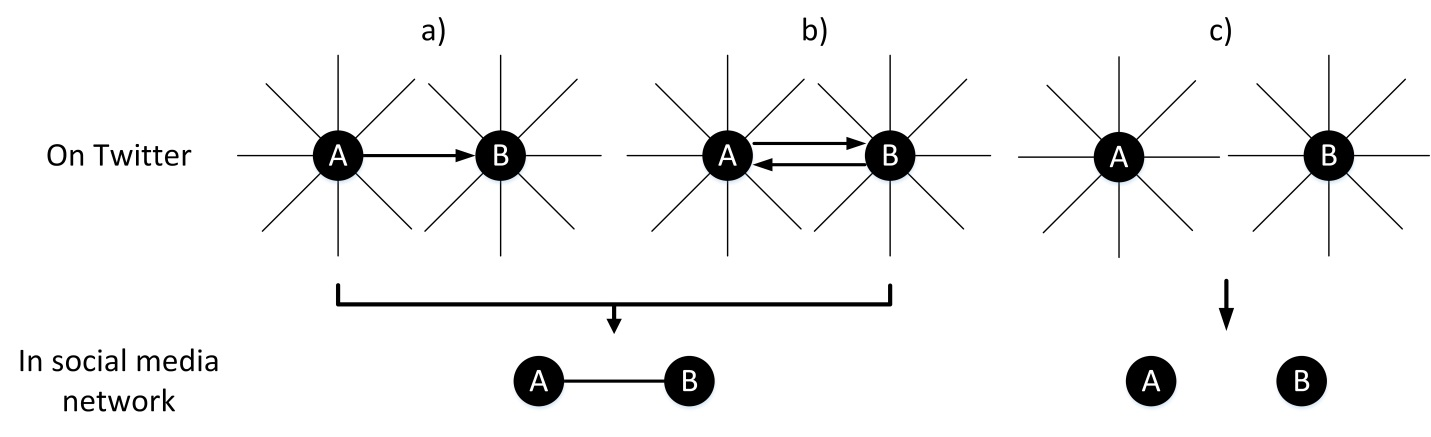

Supplement: S3 Fig — Node A and node B represent two Twitter users. Stubs without arrows represent public tweets, and lines with arrows represent replying tweets. An undirected link exists between A and B if a) A replied to B or vice versa; or b) A and B replied to each other. If c) no replying tweet exists between A and B, A and B are not considered connected in the social media network. (TIF) [file pone.0167267.s004.tif]

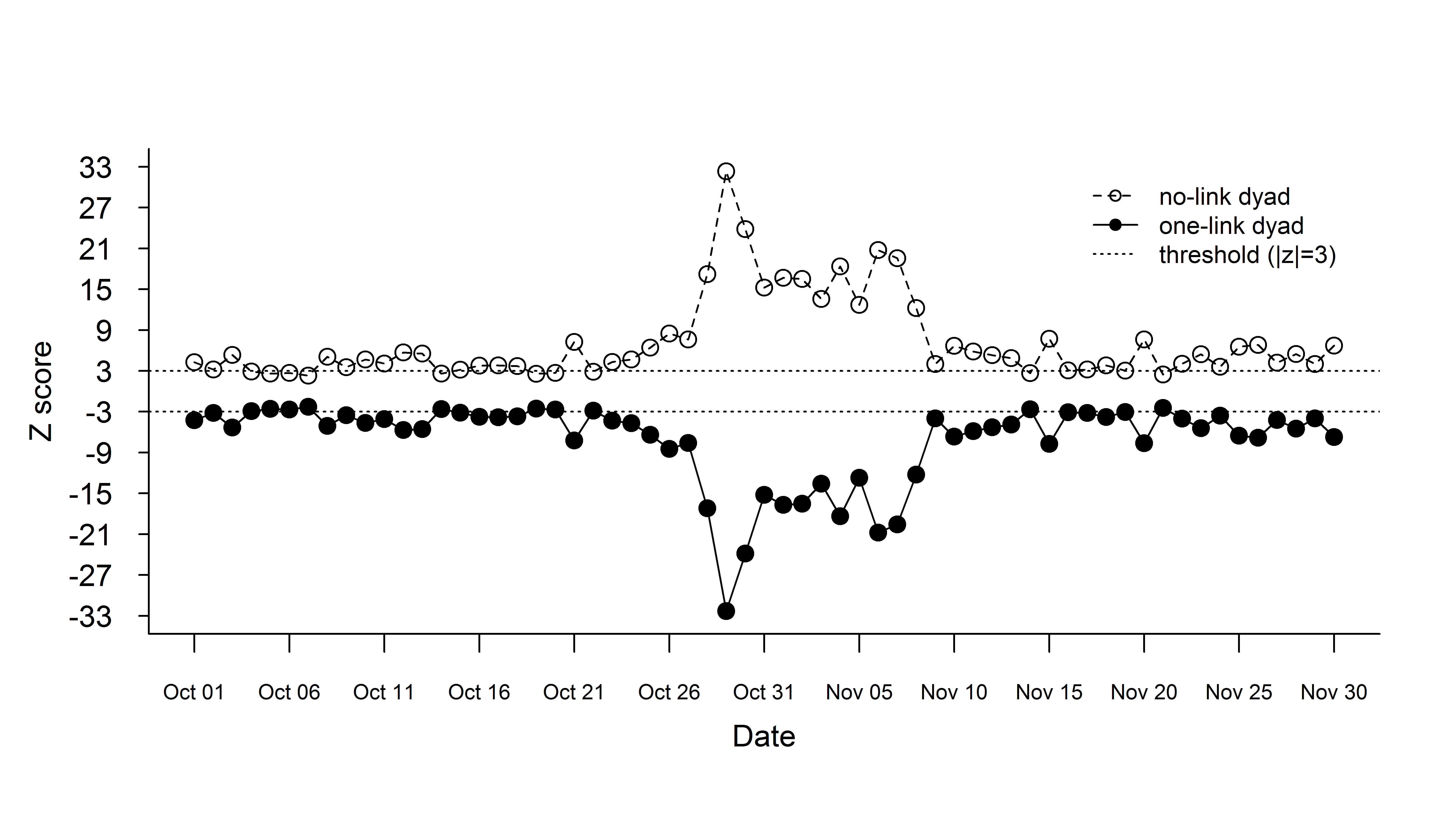

Supplement: S4 Fig — Similar with scale-free networks, the controlled property in this case is the degree distribution. The differences are that the degree distribution is assumed to be exponential in a Erdős–Rényi random graph and power-law in a scale-free network. (TIF) [file pone.0167267.s005.tif]
